# The Infectious Danger of Corticosteroids: A Fatal Case of Pneumocystis Jirovecii Pneumonia in a Non-HIV Patient Following Corticosteroid Use with Prophylaxis

**DOI:** 10.7759/cureus.5874

**Published:** 2019-10-09

**Authors:** Megha Jagannathan

**Affiliations:** 1 Internal medicine, Wayne State University School of Medicine, Detroit, USA

**Keywords:** immunocompromise, corticosteroids, pneumocystis jirovecii pneumonia (pjp)

## Abstract

Pneumocystis jirovecii pneumonia (PJP), historically regarded as an AIDS-defining illness, has been increasingly reported in non-HIV patients due to a myriad of risk factors resulting in immunosuppression. One of the more salient risk factors is corticosteroid use, including both low and high doses in prolonged, short-course, and intermittent-course regimens. The stance on PJP prophylaxis with trimethoprim-sulfamethoxazole (TMP-SMX) for non-HIV patients on corticosteroids alone (e.g., for inflammatory conditions) is unclear, with no official guidelines classifying patients by dosage, length of treatment, or preexisting conditions. Additionally, clinicians often prescribe significant dosages of corticosteroids without proper consideration of the immunosuppressive risk. Here, we describe a case of a non-HIV patient with suspected dermatomyositis who was initially prescribed prednisone 15 mg daily with no prophylaxis for one month, then increased prednisone 80 mg daily with added TMP-SMX prophylaxis. Three days following increase, the patient developed significant PJP-associated pneumomediastinum and expired within one week despite mechanical ventilation and aggressive TMP-SMX treatment. This deterioration within days following corticosteroid increase with appropriately prescribed prophylaxis is an unusual presentation of PJP pneumonia and emphasizes the fulminant progression of the disease. The unnecessary over-prescription of steroids in unconfirmed autoimmune conditions has led to an unfortunate increase in devastating infections such as PJP. Clinicians should maintain high clinical suspicion concerning the development of PJP pneumonia in corticosteroid patients as well as consider prophylaxis even before a significant steroid dose increase is prescribed.

## Introduction

Pneumocystis jirovecii pneumonia (PJP) in non-HIV patients has been steadily increasing, assumed to be due to the prescription of immunosuppressive medications in the treatment of hematologic/solid organ malignancies and autoimmune conditions (rheumatoid arthritis, etc.) [1]. In the case of autoimmune conditions, the threshold is low for prescribing corticosteroids in the appropriate clinical picture - e.g., a muscle biopsy is not required before beginning steroid therapy for clinically classic myositis. Thus, increased unnecessary corticosteroid use has resulted in significant cases of PJP in the non-HIV population, primarily attributed to steroid-induced immunosuppression.

The phenomenon of corticosteroid use predisposing to PJP has been demonstrated in doses as small as 16 mg in short course (≤4 weeks) and prolonged course (>4 weeks) regimens as well as intermittent non-daily use [2,3]. Prophylaxis with trimethoprim-sulfamethoxazole (TMP-SMX) has been shown to be highly effective in preventing PJP infection in the non-HIV immunosuppressed population, reducing occurrence rates by up to 85%; however, no universal guidelines dictate when to definitively begin prophylaxis in this patient subset [4,5].

In the HIV population, CD4+ count < 200 cells/uL has been identified as a salient risk factor necessitating PJP prophylaxis [6]. In non-HIV patients, the guidelines dictate several situations in which TMP-SMX prophylaxis should be offered: 1) patients receiving prednisone ≥ 20 mg daily for >1 month in conjunction with another source of immunocompromise, 2) patients with acute lymphoblastic leukemia, 3) patients on alemtezumab, temozolmide/radiotherapy, idelaisib, or purine analog/cyclophosphamide, 4) patients with primary immunodeficiencies, and 5) patients who received a hematopoietic stem cell or solid organ transplant [7-9]. Though many clinicians tend to mold these guidelines into other unique situations of immunosuppression, no stable guidelines have been defined on when to begin prophylaxis in corticosteroid-alone patients. The relationship between the extent of leukopenia and necessity of prophylaxis in these non-HIV patients has not been clearly elucidated.

Definitive diagnosis of PJP in the HIV and non-HIV population is through identification of cystic or trophic forms of the organism in respiratory secretions via Pneumocystis-specific stains [5]. Elevated 1-beta-D-glucan, a component of Pneumocystis cell walls, is also highly suggestive of PJP (but is not diagnostic) [10]. An additional diagnostic challenge in the non-HIV population is the tendency to have less PJP organisms demonstrated in secretions despite more severe disease course [11].

## Case presentation

History of present illness

This patient was a 55-year-old African-American male with a recent hospital admission for hypoxia/cough/dysphagia who presented to the intensive care unit (ICU) after he developed an O_2_ desaturation to 70%, tachypnea, and tachycardia while being evaluated in a positron emission tomography (PET) scanner for suspected malignancy. He was taken to the emergency department and placed on non-rebreather improving his O_2 _saturation to the 90's; however, he reported odynophagia and increased work of breathing, and was subsequently intubated and transferred to ICU. Due to suspicion of inflammation, the ICU team began broad-spectrum antibiotics of cefepime, metronidazole, vancomycin, and azithromycin as well as obtained respiratory cultures.

During previous hospitalization one month earlier, the patient presented with nonspecific ascending right hand weakness up to the shoulders and periorbital edema for a duration of three months. At that time, he was extensively evaluated for infectious etiology and autoimmune etiology, and was found to have elevated C-reactive protein and erythrocyte sedimentation rate. Additionally, he had positive rheumatoid factor, anticyclic citrullinated peptide, anti-nuclear antibody and anti-Sjögren's syndrome-related antigen A, with normal creatine phosphokinase, aldolase and myomarker myositis panel. He was thus begun on prednisone 15 mg daily by rheumatology for suspected dermatomyositis or polymyalgia rheumatica, and instructed to follow up outpatient.

**Figure 1:**
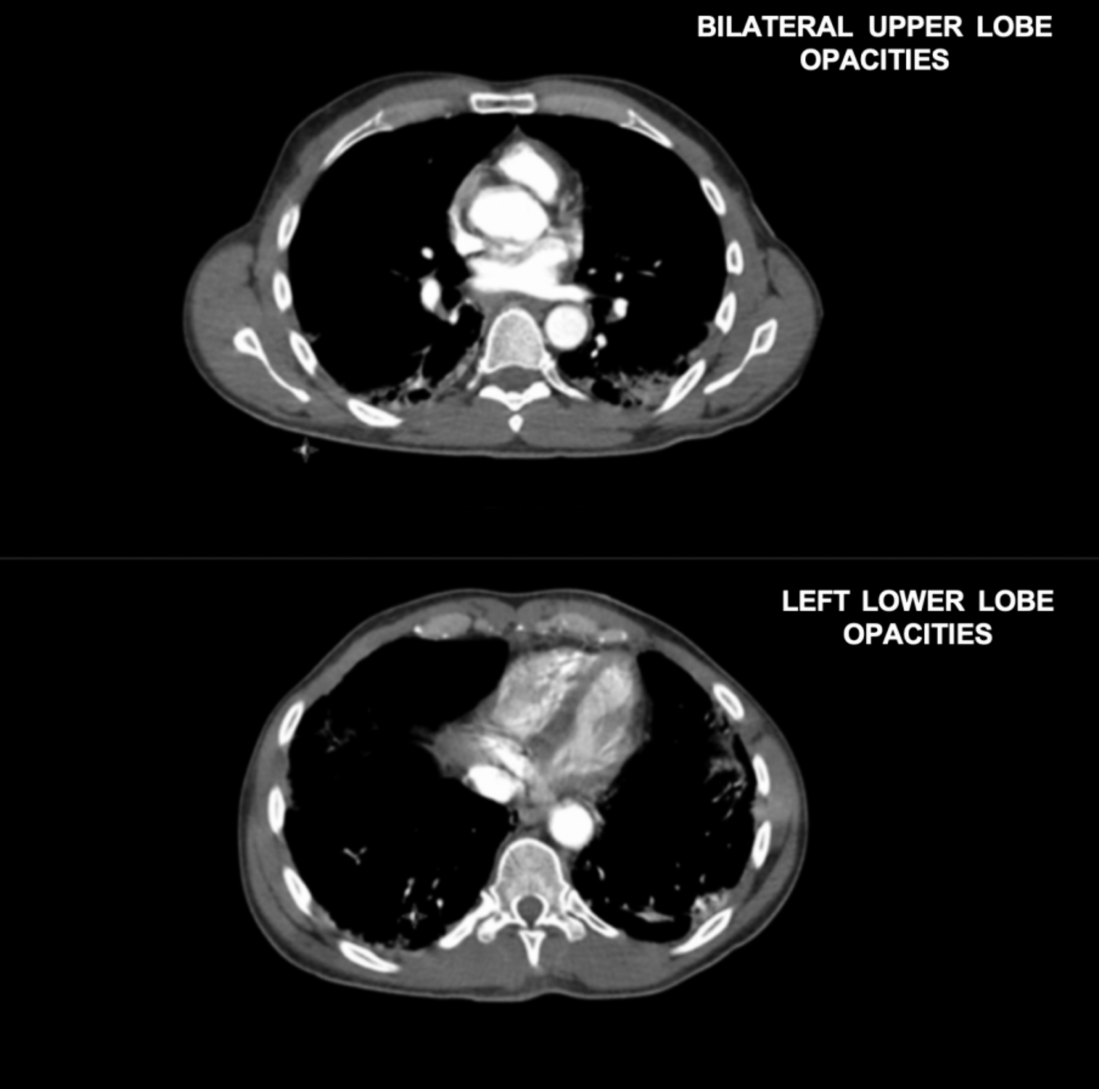
CT from hospital admission one week ago demonstrating focal scattered ground-glass and interstitial airspace opacities in the bilateral upper lobes and left lower lobe, which may have represented early pneumonic infiltrates.

Three weeks later, the patient was again hospitalized with a dry, nonproductive cough for months, hoarseness, odynophagia, dysphagia to solid and liquids, weight loss of 30 lbs, and fatigue. CT thorax demonstrated focal scattered ground-glass and interstitial airspace opacities in the bilateral upper lobes and left lower lobe, which may have represented early pneumonic infiltrates (Figure 1). During that admission, he received one dose of azithromycin and ceftriaxone in the emergency department. The patient’s O_2 _saturation was in the 90’s. However, at that time, infectious disease was not consulted, antibiotics were discontinued, and no infectious workup was completed due to suspicion of interstitial lung disease related to his autoimmune condition as opposed to infectious etiology. Ear, nose, and throat (ENT) service evaluated him for dysphagia/odynophagia and recommended PET scan outpatient for suspected malignancy. Neck CT was benign. Rheumatology evaluated him for his autoimmune condition, and increased his prednisone to 80 mg daily due to assumed autoimmune progression as well as prescribed TMP-SMX 160 mg daily for PJP prophylaxis. He was subsequently discharged and obtained his PET scan one week later where he experienced this marked desaturation.

Past medical history included the aforementioned suspected dermatomyositis, bilateral hip degenerative joint disease on corticosteroid injections status post total hip replacements, dementia, and previous acute kidney injury. Past surgical history included bilateral total hip replacements three years prior. No known drug allergies were reported. Medications at home included prednisone 80 mg daily, trimethoprim-sulfamethoxazole (TMP-SMX) 160 mg daily for PJP prophylaxis, pregabalin 200 mg twice daily, meloxicam 15 mg daily, celecoxib 200 mg daily as needed for pain, Vitamin D3 1000 IU daily and pantoprazole 40 mg daily. Social history was obtained through family members. He had an unknown amount of cigarette usage and no alcohol, marijuana, or recreational drug history. The patient had no recent travel or sick contacts. He worked in construction, and consistently wore a mask during renovations.

Physical examination and initial imaging

**Figure 2:**
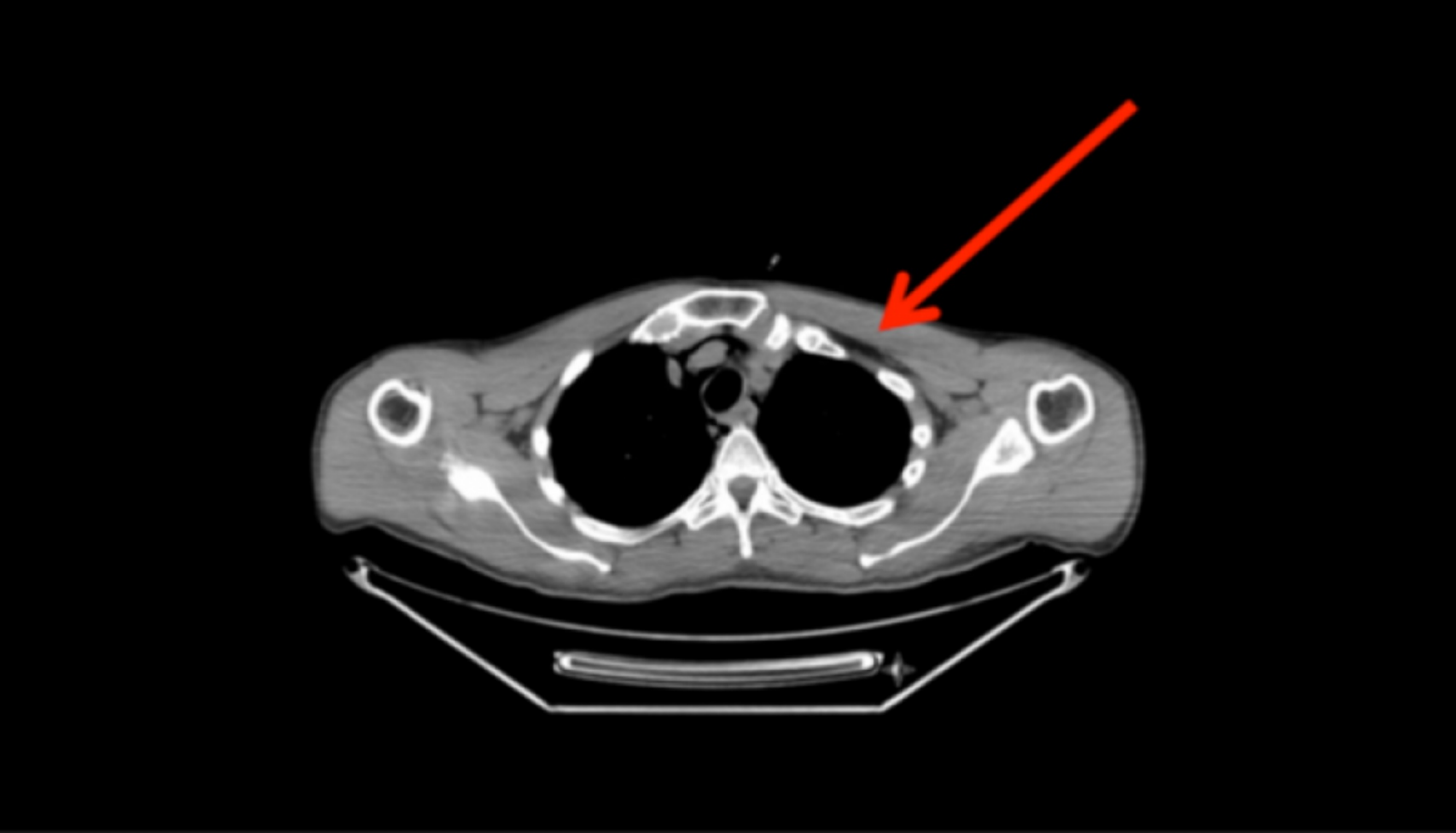
Positron emission tomography (PET) scan demonstrating significant pneumomediastinum up to lower neck.

**Figure 3:**
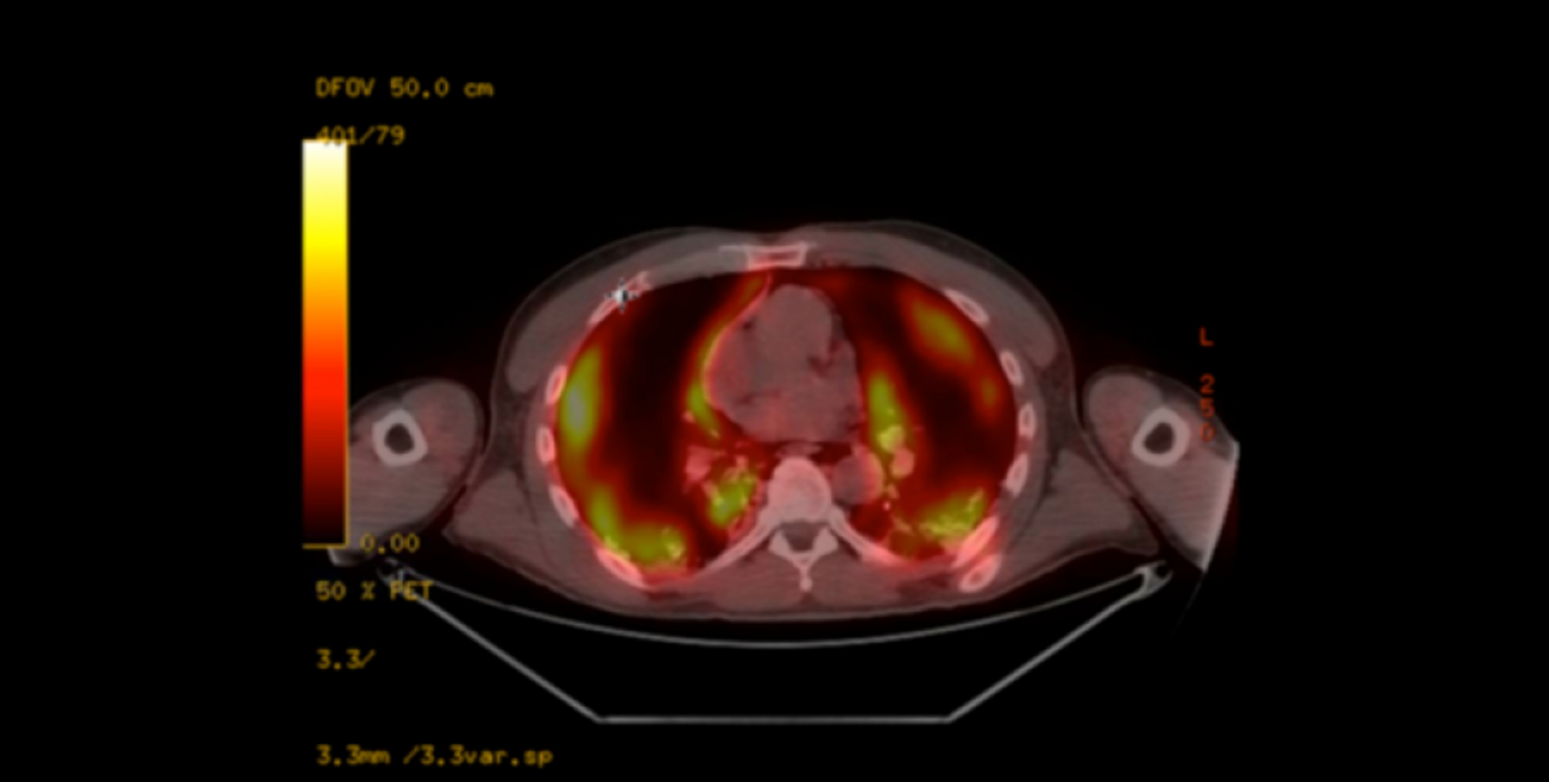
Positron emission tomography (PET) scan demonstrating increased uptake bilaterally in areas with ground-glass opacities.

Vitals in the PET scanner showed blood pressure of 115/80 mmHg, heart rate of 122 bpm, respiratory rate of 28/minute, temperature of 36.8°C, and O_2_ saturation in the 70s. The patient was subsequently intubated and placed on mechanical ventilation with high positive-end-expiratory-pressure (PEEP), with settings tidal volume 500 ml, respiratory rate 28/minute, fraction of inspired O_2_ of 100%, and PEEP of 10 cmH_2_O. The patient was sedated with fentanyl and midazolam. Bronchoalveolar lavage was also obtained. PET scan results revealed bilateral patchy ground-glass opacities with increased uptake representing an inflammatory etiology as well as extensive pneumomediastinum extending into the lower neck (Figures 2, 3). Physical exam revealed bilateral scattered crackles in both lung fields. No other pertinent positives were noted.

Laboratory studies

Before intubation and transfer to ICU, labs showed an arterial blood gas (ABG) of pH 7.45 (ref. 7.35-7.45), pCO_2_ 28 mmHg (ref. 36-45), pO_2_ 77 mmHg (ref. 80-100), HCO_3_^-^ 22.4 mEq/L (ref. 22-28). Basic metabolic panel revealed mild hypocalcemia at 8.5 mg/dL (ref. 8.6-10.8) with all other values within normal limits. Complete blood count values were all within normal limits, with white blood cells (WBC) at 7.1 K/CUMM (ref. 3.5-10.6) and normal differential, hemoglobin (Hb) at 15.0 gm/dL (ref. 13.3-17.1), and platelets at 396 K/CUMM (ref. 150-450). HIV Ag/Ab was nonreactive, and HIV-1 RNA was undetectable. Decreased CD4+ count to 164 cells/uL (ref. 580-1929) and CD8+ count to 18 cells/uL (ref. 359-1199) was demonstrated; however CD4:CD8 ratio was 2.70 (ref. 0.98-3.24), consistent with pan-immunosuppression.

Viral panel was negative. Fungal, legionella, mycobacteria culture were negative. Streptococcus pneumonia and flu were negative.

The following day after intubation, the patient’s bronchoalveolar lavage revealed positive Pneumocystis organisms on Pneumocystis stain and infectious disease was consulted. Lactate dehydrogenase and Beta-D glucan were also significantly elevated at that time to 520 units/L (ref. 140-271) and 445 pg/mL (ref. 0-59).

Treatment and clinical course

**Figure 4:**
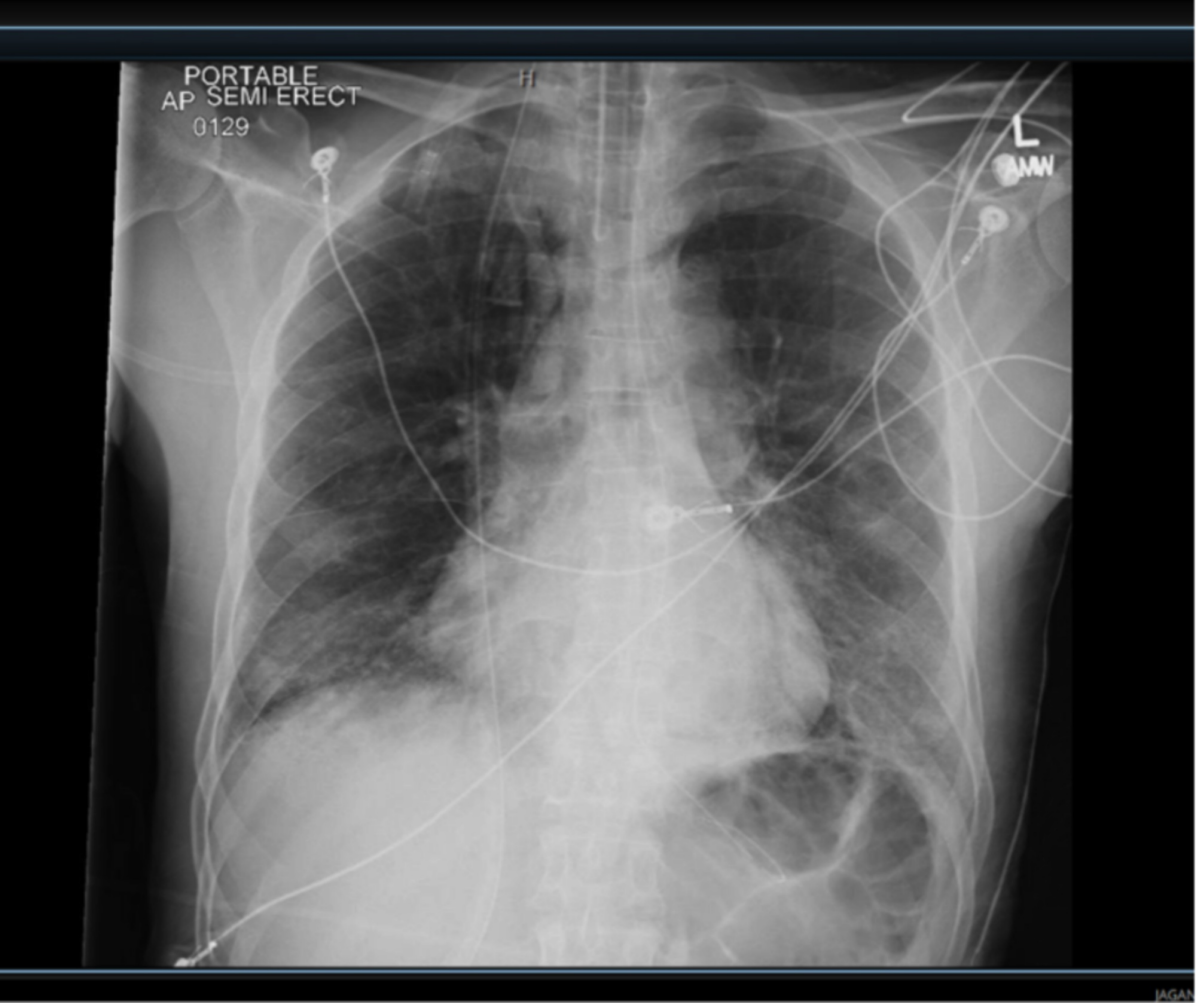
Chest X-ray (CXR) one day post admission demonstrating pneumomediastinum.

**Figure 5:**
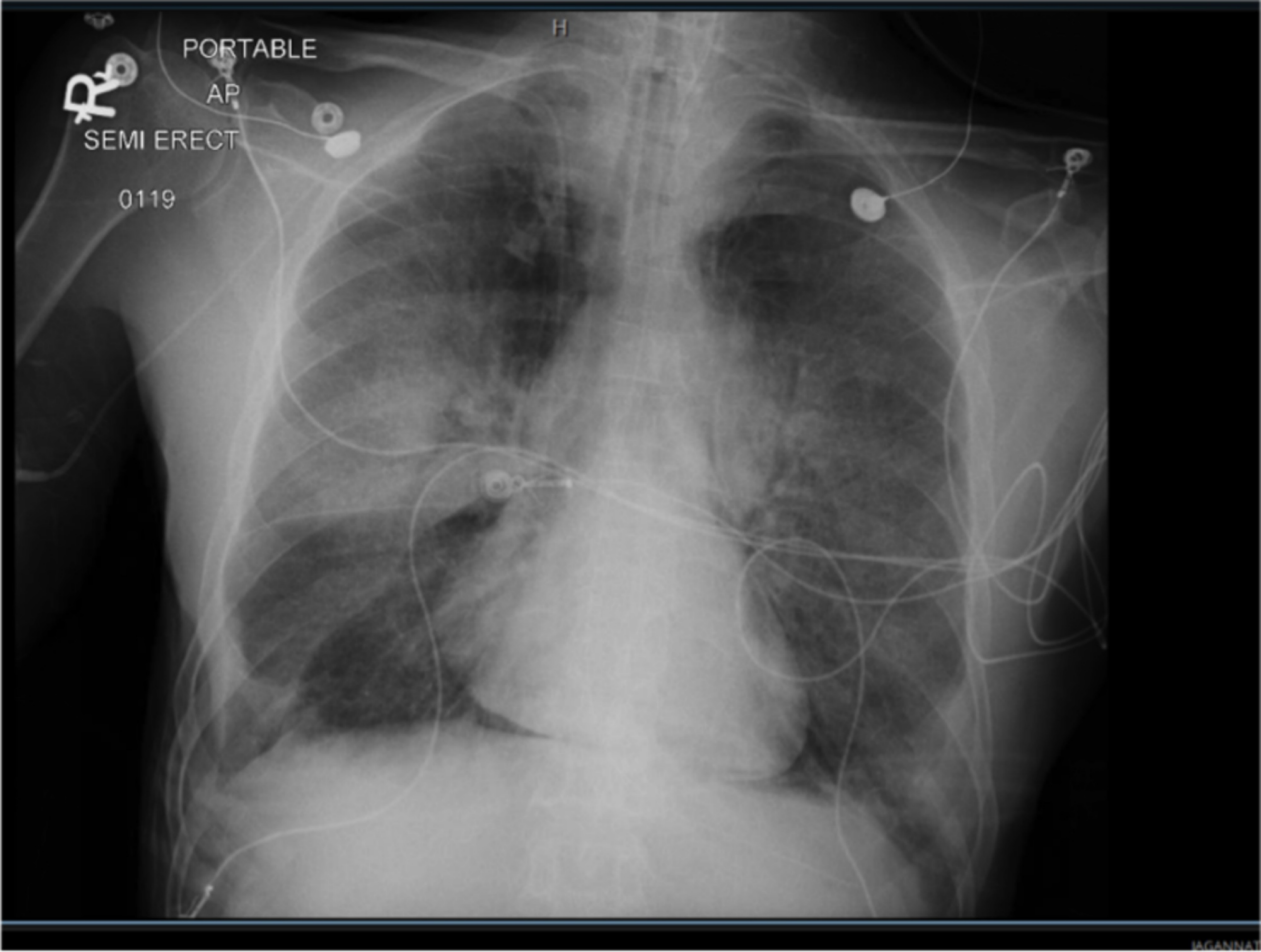
Chest X-ray (CXR) two days following TMP-SMX demonstrating worsening pneumomediastinum and infiltrates. TMP-SMX: Trimethoprim-sulfamethoxazole

The following day after intubation, chest X-ray revealed worsening pneumomediastinum and PEEP was reduced to 8 cmH_2_O (Figures 4, 5). Upon positive Pneumocystis stain, the patient’s previous antibiotics were stopped and he was started on TMP-SMX 320 mg every eight hours and methylprednisolone 40 mg twice daily for PJP (in total, two days following admission). Despite treatment, the patient’s pneumomediastinum on chest X-ray gradually worsened over the subsequent days. The patient continually required increasing PEEP to saturate appropriately, up to a PEEP of 15 cmH_2_O. WBCs were continually increasing, from 9 → 13 → 24 K/CUMM. ABGs were gradually worsening, demonstrating increased acidosis and decreased O_2 _saturation. The patient’s family subsequently decided to sign a do not resuscitate order due to his deteriorating condition. The following night (six days following admission and four days post TMP-SMX initiation) the patient went into respiratory failure and expired.

## Discussion

PJP in the non-HIV population is particularly challenging; it presents with a more fulminant course and possesses higher mortality rates of 30-50% [1,3]. Additionally, PJP in non-HIV patients demonstrates higher ICU admission rates with need for ventilatory support compared to its HIV counterpart [4]. Overall, while HIV-associated PJP presents with exuberant hypoxemia and lung inflammation, PJP in the non-HIV population presents with insidious dry cough rapidly deteriorating to profound respiratory failure [5].

This case presented a particular challenge in PJP management due to significant rapid onset despite initiation of prophylaxis as well as subsequent treatment failure with development of respiratory decompensation. The patient’s previous CT scan one week prior demonstrating ground-glass opacities most likely demonstrated early PJP as a result of previous low-dose prednisone usage without prophylaxis. His dry cough and discomfort also most likely represented early PJP infection. However, it is still unclear whether this patient developed PJP before prophylaxis administration, or developed PJP-induced respiratory failure as the result of the dose-dependent immunosuppressive effect of increased corticosteroids. Additionally, although rare, this case could have potentially represented an instance of TMP-SMX resistance.

This case also highlighted the significant dangers associated with inappropriate steroid prescription in patient care. The sole predisposing factor to PJP development in this patient was corticosteroid use based on clinical suspicion of myositis with no tangible proof. Treatment of immunologic conditions is certainly a critical aspect of patient management; however, in this case, the risk of corticosteroid use far outweighed the potential benefit (which was ultimately not demonstrated in this patient before his death). The steroid increase amplified the progression of this patient’s respiratory infection and the suspected myositis itself was not rectified with the steroids, highlighting a lapse in judgment in the free-handed prescription of corticosteroids.

## Conclusions

Further research is needed to establish definitive guidelines of PJP prophylaxis in the non-HIV population, as well as when to initiate empiric treatment in the setting of suspected pneumonia. Immunosuppression due to corticosteroid use is a salient predisposing factor in the development of PJP in an aggressive and debilitating course, and physicians should maintain high levels of discretion in significant steroid prescription for minor conditions. Clinicians should ultimately have a very low threshold for infectious disease consultation and management in the case of non-HIV immunosuppressed patients presenting with pneumonia symptoms. Earlier prophylaxis or treatment could have prevented this patient’s rapid deterioration, and prevention of similar cases is possible with intense discretion in steroid prescription as well as early aggressive intervention.
